# Making the case for service user involvement in the operating department practice curriculum: A discussion paper

**DOI:** 10.1177/17504589241302940

**Published:** 2025-03-26

**Authors:** Emil Siwadi, Mel Hughes

**Affiliations:** 1 Operating Department Practice, Bournemouth University, Bournemouth, UK; 2 Social Work and The PIER Partnership (Public Involvement in Education and Research), Bournemouth University, Bournemouth, UK

**Keywords:** Service user, Operating department practice, Education, Humanisation

## Abstract

This article discusses the case for improving service user involvement in a UK-based operating department practice (ODP) degree programme. The term ‘service user’ is a broad phrase referring to those who use or are affected by the services of Health and Care Professions Council-registered professionals. In 2018, the Health and Care Professions Council set a mandatory requirement for service user engagement within all 15 HCPC-registered Allied Health Professions. Despite this, there is a lack of published literature on this topic, particularly in relation to operating department practice education. The authors, who are involved in operating department practice education and service user engagement at one UK university, discuss their proactive integration of service user involvement in the operating department practice curriculum through a well-established partnership. The article identifies various formats of service user involvement, utilisation of a humanisation framework, evaluating the impact on students and highlights the transformative potential of experiential learning. The authors advocate for knowledge sharing to build an empirical foundation for service user involvement in operating department practice education nationwide.

Operating department practices (ODPs) are an essential part of the multidisciplinary operating theatre team and provide patient care through the assessment, planning and delivery of individualised care to maintain or restore the physiological and physical status of the perioperative patient at all levels of dependency. Although primarily employed within operating theatres, ODPs are increasingly being recognised for their skills in other clinical areas.

ODPs are Allied Health Professionals (AHPs) and in the United Kingdom are registered with the Health and Care Professions Council (HCPC). In 2012, HCPC carried out a consultation to see if service user engagement should become a mandatory requirement in the standards of education and training (SETs) (HCPC 2012). HCPC guidance (HCPC 2012) encouraged programme providers to evidence service user involvement in their programmes, such as through service user feedback in monitoring and evaluating programmes (SET 3.3) and the contribution of service users to teaching and learning (SET 4.8), but service user involvement was not a mandatory requirement, and it was possible for a programme to be validated without any service user involvement. In addition, the Professional Standards Authority for Health and Social Care (PSA), who regulate nine regulators in their remit, one of whom is the HCPC, expects service users to be involved in the delivery of approved programmes (PSA 2019). HCPC (2018) uses the term ‘service user’ as an umbrella term to refer to those who directly use or are affected by the services of professionals registered with the HCPC. In September 2018, the revised HCPC SETs came into effect (HCPC 2017) with a new standard that made the requirement for service user involvement mandatory in all HCPC-approved programmes. HCPC standards are regularly reviewed. As part of the latest review, five key themes were highlighted in an HCPC webinar (Campbell 2023, 08:39), including the need to centralise the service user in health care education. Within this, there were a number of key aims presented, in particular, registrants are required to ‘think broadly about [service users] best interests . . . provide care that upholds their rights, dignity, values and autonomy in an appropriate and effective way’ and ‘be mindful of the needs of people living with a disability or a health condition which impacts their ability to communicate’ (Campbell 2023). A 2020 Community Research report stated that service users felt that the revised HCPC standards ‘were a step in the right direction in ensuring inclusive practise and empowering all service users’ (Community Research 2020: p12). Although the standards relate to registered practitioners, HCPC-approved programmes are designed to ensure graduates can meet the requirements of a registered practitioner and summative assessments link directly to the standards. The HCPC has not been prescriptive about who service users and carers are; instead, they state the need to be satisfied that programmes have chosen the most appropriate and relevant service user groups for that programme and profession (Campbell 2023). The deliberate non-prescriptive approach to defining the term ‘service user’ is in line with several other key standards recently published by the National Institute for Health and Care Research (NIHR 2022), namely, the Patient and Public Involvement and Engagement Resource Pack and The UK Standards for Public Involvement (2019). Although NIHR focuses on service user involvement with health care research, they emphasise a shift in health care development that seeks service user involvement that goes beyond merely recruiting participants for studies but instead includes engaging service users in shaping research agendas, designing studies and interpreting results (NIHR 2022). NIHR emphasises that service user involvement should be meaningful and embedded at various stages of research, ensuring that the research is relevant to those who will be affected by its outcomes, ensuring that research addresses real-world needs and improving health and care services based on the perspectives and experiences of those directly impacted (NIHR 2022). The authors believe this ethos should also be at the forefront of meaningful service user involvement within health care undergraduate education, to ensure service user involvement leads to better quality care in practice.

Despite the requirement for mandatory service user involvement, a scoping literature search by the authors returned only one paper, by Richardson et al (2013), which focused on service user involvement in ODP interviews. A further scoping literature search, by the authors, for service user involvement in other AHP degree programmes returned a small number of published papers, particularly around physiotherapy undergraduate education. This is in stark difference to the extensive literature available in relation to involvement in nursing, social work and medical education. This could be due to the fact that ODP is almost exclusively a UK-based profession. The title ‘ODP’ is not widely used globally, and the role of registered theatre practitioners varies across the world. Many countries employ anaesthetic or scrub-specific practitioners, who are trained to specialise in one area of perioperative practice only.

Although the HCPC SETs have applied to all ODP programmes since 2018, evidence of the impact of service user involvement in ODP education is distinctly lacking. It is unclear if this is due to a lack of activity, an absence of reporting or a low value placed on service user involvement in ODP education. As identified in relation to physiotherapy education (Jury et al 2023), the lack of published literature does suggest the absence of a well-developed culture and framework for high-quality, evidence-informed service user involvement activity in ODP education in the United Kingdom. This needs to be addressed.

As academics based in the Faculty of Health and Social Science at a Higher Education Institution (HEI) in the South of England, who are active members of a service user involvement in education and research partnership, the lack of published literature led us to acknowledge that despite having embedded lived experience expertise in our ODP programme, we had not reported or published this work outside of our own faculty.

A lack of published literature may create an additional challenge for education providers wishing to assess what works and who it works for. Also, the ODP role is not widely understood by those outside of the profession, and service users may struggle to see where they could be involved in ODP education.

A scoping review, by Sturgiss et al (2022), looking at how patient-centred care has been represented in health care literature, found that the majority of evidence was from the United States, where the ODP profession does not exist, and that, globally, nursing was the most commonly represented profession. Another key finding was that the service user perspective was rarely included, with only 15% of the papers reviewed (Sturgiss et al 2022).

The current HCPC SETs stipulate that service users must be involved in the programme (SET 3.7), without stipulating how this might be achieved. Being non-prescriptive allows HEIs a broad scope to decide which service user groups are best suited to engage with and effectively embed service user involvement in ODP programmes; however, this lack of directed practice could lead to different levels of quality. A study by Read et al (2020), which included a broad range of health care programmes, at a Midlands-based university, indicated the importance of co-production, between service user groups and academics, to design, deliver and evaluate programmes to ensure the desired outcomes were achieved.

At our university, we have a well-established and active partnership with a service user organisation, called Public Involvement in Education and Research (PIER), who understand the ODP role, having undergone surgical procedures, working with the ODP programme team and engaging with our ODP students throughout the degree programme. The partnership was first established in 2005 within the faculty’s social work programme and now coordinates over 1000 hours of direct involvement between people with lived experience, students and academics each year, across multiple health and social care programmes including social work, occupational therapy, adult, mental health and children’s and young people’s nursing, paramedic science, physiotherapy and midwifery. Within the faculty, colleagues from other AHP programmes have shared their experiences of embedding service user involvement in their programmes, allowing the ODP programme team to understand activities that have worked for them.

Service user involvement can take place in a range of formats or spectrums. Towle et al (2010) defined the spectrum of service user involvement as having six main educational roles:

- Paper-based scenario.
- Simulated patient.
- Patient sharing experiences with staff facilitating.
- Patient teacher – teaching or evaluating.
- Patient teacher as partner in design, delivery and evaluation of curriculum.
- Involved at institutional level.

The humanisation framework (Todres et al 2009) has been embedded within the ODP programme since 2019, when structured PIER activities and principles of humanisation were written into the indicative content for specific units and offer a foundation on which to embed lived experience expertise by integrating the theoretical concept of humanisation with service users to explore the lived experience.

Within our ODP programme, students take part in structured discussion/group work with service users, giving students the opportunity to explore the principles of humanisation and link them directly to their own experiences of practice and the relation with patient-centred care. During these sessions, individual dimensions of the humanisation framework (Figure 1) were explored and brought to life, through hearing about and discussing the individual experiences of PIER members, further offering students the opportunity to link and make strong correlations between theoretical concepts and real-world clinical practice. The eight dimensions of humanisation are intertwined emphases; each emphasis has a positive humanising element as well as a related negative dehumanising emphasis. Having the opportunity to explore these dimensions with service users can enable a deeper understanding of not only how our professional actions or lack of action can enhance wellbeing or foster negative emotions and dehumanise care.

**Figure 1:**
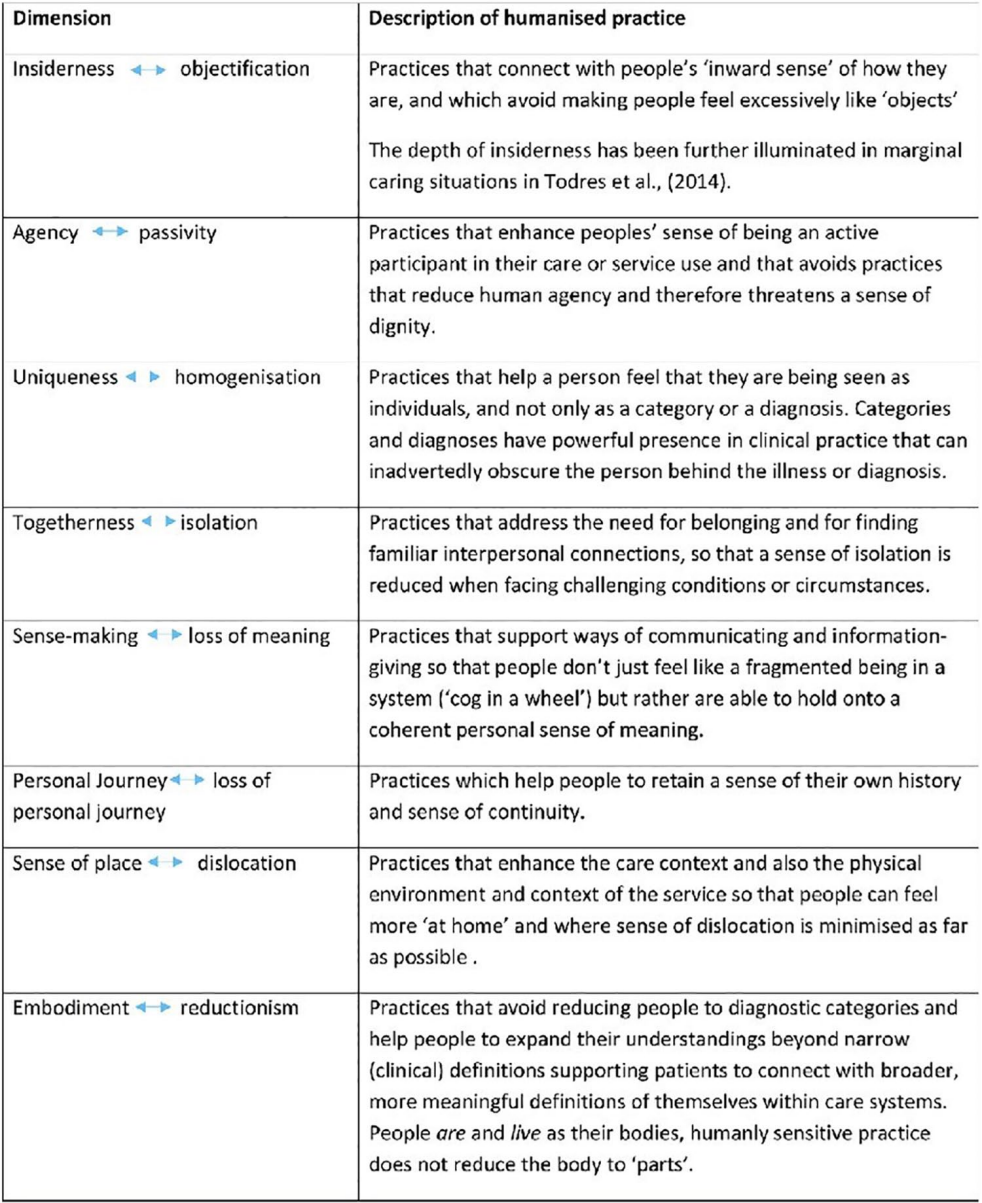
The humanisation framework (Galvin et al 2016, 2020, Karlsson et al 2019) reproduced with kind permission from Professor Kathleen Galvin

Within our own institution, PIER activity includes: contributing to lectures; creating digital resources such as real-life case studies and interviews; assessing students’ work by being presentation assessment panels or providing feedback on portfolios; codesigning the curriculum alongside academics, students and practitioners, facilitating groups; contributing to book chapters; and engaging in simulation and role-play. When evaluating the impact of this involvement, and the impact of different types of activity, students undertaking in-class activities involving people with lived experience *of a range of long-term health conditions, substance use, disease and mental or physical impairment, across the faculty, are routinely asked to complete a simple evaluation form at the end of each activity. As part of an internal review, a thematic analysis* (Braun & Clarke 2006) *was conducted by the PIER team on 3663 evaluations of 142 activities delivered across eight professional programmes (social work, adult nursing, mental health nursing, children and young people’s nursing, midwifery, physiotherapy, occupational therapy, paramedic science)* over a 2-year period between 2017 and 2019. Six themes were identified from the results. Four related to the meta-theme: types of learning (increased knowledge, ways of improving practice; enhanced emotional resilience and meeting professional requirements) and two related to the meta-theme: approaches to involvement (opportunities for discussions and opportunities to gain feedback). The internal study showed that learning is enhanced when the nature of the involvement includes discussions between students and people with lived experience, and when there are opportunities for students to gain feedback on their own skills and developing practice. This enables students to engage with the subject and gain support from experts by experience to apply it to their own practice. There is evidence that the learning can be transformative.

The PIER partnership supports teaching across all 3 years of the ODP programme. Although COVID restrictions, and the need to protect clinically vulnerable members of the partnership, did have a short-term impact on face-to-face delivery (when all PIER activity moved online), these sessions are now all delivered in person and are interactive. Sessions are planned in advance, allowing the opportunity for planning meetings prior to each session. Key learning objectives, in line with the unit’s intended learning outcomes, are identified and agreed by the academic and participating PIER members. The emphasis is on codesign, so as to draw on both lived experience, academic and practice perspectives, and expertise. The sessions are guided, but students are encouraged to ask questions and explore their understanding of the lived experience. Over the years, different strategies have been used and by formally collating feedback from academics, service users and students who took part in each session, we have been able to learn what works and what has not worked. Through seeking and sharing feedback from and between all those involved in the sessions, valuable data can be gathered to better understand the value and quality of service user involvement in the curriculum. These are currently being developed as a deeper evaluation of service user involvement activities and will be published separately.

Having established service user involvement in the ODP programme since 2019, the next step will be to share our experiences and ideas for enhancing service user involvement in ODP education in the United Kingdom. This will be done by collaborating with PIER members who have engaged in the ODP programme to produce a paper that evaluates the impact of their activity on students, academics and the PIER members themselves. Our goal is to share examples of what works best to enhance learning and to contribute to a much-needed empirical evidence base for service user involvement in ODP education nationally.
